# Frequency and Risk Factors for Metastasis in Newly Diagnosed Appendiceal Carcinoma

**DOI:** 10.7759/cureus.16341

**Published:** 2021-07-12

**Authors:** Ahmed Minhas, JeanMarie Hendrickson, Sohail A Minhas

**Affiliations:** 1 Internal Medicine, East Tennessee State University’s Quillen College of Medicine, Johnson City, USA; 2 Mathematics and Statistics, East Tennessee State University’s Quillen College of Medicine, Johnson City, USA; 3 Hematology/Oncology, Baptist Memorial Hospital-Memphis, Memphis, USA

**Keywords:** appendiceal carcinoma, carcinoma of the appendix, goblet cell adenocarcinoma, signet ring cell adenocarcinoma, colonic-type adenocarcinoma, mucinous adenocarcinoma of appendix, cancer epidemiology, metastatic appendix cancer

## Abstract

Background

Appendiceal carcinoma has an insidious clinical presentation, and these tumors are rarely suspected prior to surgery, potentially leading to late diagnosis. The aim of this study is to investigate the prevalence of metastatic disease at initial presentation and potentially associated sociodemographic characteristics.

Methods

Patients were identified from the Surveillance, Epidemiology, and End Results (SEER) program using the International Classification of Diseases for Oncology-3 (ICD-O-3) histology/behavior codes between 2010 and 2015. Firth logistic regression was performed to determine the association of metastasis at presentation with tumor subtype, adjusted for age, race, sex, insurance and marital status, tumor grade, and tumor and nodal stage using the 7th edition of the American Joint Committee on Cancer (AJCC) staging system.

Results

We identified a total of 3,447 patients with known metastatic status. A total of 38.4% had metastatic disease at diagnosis. Compared to colonic-type adenocarcinoma (CA), mucinous adenocarcinoma (MA) and signet ring cell carcinoma (SC) were more likely to present with metastasis at diagnosis (OR: 2.34; 95% CI [1.80- 3.06]; OR: 1.93 [1.29-2.89], respectively), however, goblet cell carcinoma (GC) was less likely (OR: 0.59 [0.36-0.93]). Compared to tumors invading the submucosa (T1 stage), tumors invading deeper through the visceral peritoneum or nearby organs (T4 stage) were significantly more likely to present with metastatic disease (OR: 3.46 [2.24-5.51]). Tumors invading the muscularis propria (T2 stage) or deeper into the subserosa, or the mesoappendix (T3 stage) were less likely to present with metastatic disease (OR: 0.34 [0.16-0.71]); OR: 0.55 [0.34-0.91], respectively). Compared to no regional lymph node spread, four or more regional lymph node involvement (N2 stage) was more likely to present with metastatic disease (OR: 2.19 [1.53-3.16]). Men were less likely to present with metastatic disease (OR: 0.60 [0.48-0.73]). A total of 90.1% of CA, 84.2% of GC, 42.2% of MA, and 78.5% of SC patients with metastasis at diagnosis had extraperitoneal distant metastasis (M1b).

Conclusions

A significant proportion of patients with newly diagnosed appendiceal carcinoma presented with metastatic disease, concerning substantial diagnostic delay and potentiating the need for aggressive treatments. Predictors of metastatic disease included female sex, histologic subtype, and significant regional lymph node involvement. Future research should focus on earlier detection and explore tumor biology.

## Introduction

Appendiceal cancer is a very rare and potentially aggressive malignancy. It represents less than 0.5% of all GI neoplasms [1]. The age-adjusted incidence is 0.12 cases per 1,000,000 people per year [2]. Known risk factors include smoking, family history of appendiceal cancer or multiple endocrine neoplasia type 1, atrophic gastritis, female sex, and increasing age. The five-year disease-specific survival varies depending on the tumor, node, metastasis (TNM) system staging and histologic subtype. Appendiceal cancer is categorized as carcinoid tumors and carcinomas, which are both staged differently. Carcinomas include goblet cell carcinomas (GC), colonic-type adenocarcinoma (CA), mucinous adenocarcinoma (MA), and signet ring cell adenocarcinomas (SC). From 1973 to 1998, MA was the most common carcinoma subtype, followed by CA [2]. Data from more recent years are scarce.

Appendiceal carcinoma is often diagnosed incidentally, particular­ly during surgery for appendicitis, and is rarely suspected prior to surgery since symptoms are non-specific and mimic those of other diseases. This potentially leads to diagnosis at more advanced stages. The presence of metastases greatly influences the clinical course of patients with appendiceal carcinoma. Metastatic disease confers poor prognosis and entails difficult treatments [2-5]. Due to the rarity of appendiceal carcinoma, epidemiological data on metastatic disease at presentation is limited. The objective of this study is to investigate the prevalence of metastatic disease at initial presentation and potentially associated sociodemographic and tumor characteristics.

## Materials and methods

The Surveillance, Epidemiology, and End Results (SEER) program was used to collect data (SEER Regs 18 Custom Data [with additional treatment fields], Nov 2018 Sub) [6]. A total of 3,525 patients with appendiceal carcinoma were identified using site recode International Classification of Diseases for Oncology-3 (ICD-O-3) “appendix”, year of diagnosis between 2010 and 2015, and ICD-O-3 histology/behavior codes for CA (8140, 8141, 8260, 8262 8440, 8460), MA (8470, 8471, 8480, 8481), SC (8490), and GC (8243, 8245). Only a patient’s first primary tumor in the database that matched these selection criteria was included. Patients excluded were those who were alive with no survival time and death certificate only or autopsy only cases. Tumors that were not microscopically confirmed were excluded. Of 3,525 patients with appendiceal carcinoma that were identified from 2010 to 2015, 78 patients had missing metastatic status and were excluded from the study.

Demographic and clinical data included age at diagnosis, cancer stage, marital status, race, sex, tumor grade, and subtype. Marital status was categorized into married (including common law) and not married, which included divorced, never married, separated, widowed, and unknowns. The American Joint Committee on Cancer (AJCC) TNM staging system, 7th edition, was used.

Due to the rarity of events being analyzed, firth logistic regression was used to determine the association of metastasis at diagnosis with tumor subtype, adjusted for age, insurance and marital status, nodal stage, race, sex, and tumor grade and stage. Analysis was performed using the R software with the *Logistf* package [7,8]. The East Tennessee State University institutional review board (IRB) determined that this study neither meets the US FDA nor the Department of Health and Human Services' definition of human subjects research, thus it does not fall under the purview of the IRB.

## Results

Cohort

The baseline characteristics of the 3,447 patient cohort are shown in Table 1. A total of 1,323 patients, or roughly 38.4%, had metastatic disease at diagnosis. The median age at diagnosis was 59 and 61 years for those with and without metastatic disease, respectively. MA was the most common subtype followed by CA. SC had the highest percentage of metastatic disease at presentation, followed by MA.

**Table 1:** Baseline characteristics of patients diagnosed with appendiceal carcinoma, 2010-2015.

	All patients with appendiceal carcinoma (n = 3447)	Patients with metastatic disease (n = 1323)	Histologic subtype			
			Colonic Adenocarcinoma (n = 814)	Goblet Cell Adenocarcinoma (n = 680)	Mucinous Adenocarcinoma (n = 1621)	Signet Ring Cell Carcinoma (n = 332)
Variable	Number of patients	Number of patients (% of total in variable category)	Number of patients			
Age						
<40	257	101 (39%)	45	59	135	18
40-59	1438	601 (42%)	274	324	685	155
60-79	1460	546 (37%)	375	258	692	135
≥80	292	75 (26%)	120	39	109	24
Sex						
Female	1828	784 (43%)	390	350	894	194
Male	1619	539 (33%)	424	330	727	138
Race						
Asians and American Indians	261	111 (43%)	61	29	149	22
Black	364	133 (37%)	108	70	150	36
White	2805	1074 (38%)	642	577	1313	273
Unknown	17	5 (29%)	3	4	9	1
Marital Status						
Married	2039	812 (40%)	481	369	992	197
Unmarried	1241	464 (37%)	301	264	556	120
Unknown	167	47 (28%)	32	47	73	15
Insurance Status						
Insured	3237	1249 (39%)	764	624	1529	320
Uninsured	132	54 (41%)	33	32	60	7
Unknown	78	20 (26%)	17	24	32	5
Grade						
I	831	304 (37%)	120	131	572	8
II	1136	364 (32%)	430	109	583	14
III	532	271 (51%)	147	57	133	195
IV	92	52 (57%)	9	9	39	35
Unknown	856	332 (39%)	108	374	294	80
Histologic Type						
Colonic Adenocarcinoma	814	220 (27%)	-	-	-	-
Goblet Cell Adenocarcinoma	680	68 (10%)	-	-	-	-
Mucinous Adenocarcinoma	1621	839 (52%)	-	-	-	-
Signet Ring Cell Carcinoma	332	196 (59%)	-	-	-	-
T Stage						
T0	20	20 (100%)	5	0	15	0
T1	224	40 (18%)	70	59	91	4
T2	226	14 (6%)	81	74	65	6
T3	1063	121 (11%)	288	407	284	84
T4	1611	934 (58%)	305	118	978	210
Tis	35	0 (0%)	12	6	17	0
TX	268	194 (72%)	53	16	171	28
N Stage						
N0	2523	817 (32%)	539	574	1263	147
N1	432	193 (45%)	148	61	145	78
N2	260	157 (60%)	85	24	64	87
NX	232	156 (67%)	42	21	149	20
M Stage						
M0	2124	-	594	612	782	136
M1a	533	-	20	9	465	39
M1b	712	-	183	48	339	142
M1, not otherwise specified	78	-	17	11	35	15

Sociodemographic and tumor characteristics associated with metastatic disease at diagnosis

Table 2 shows the sociodemographic and tumor characteristics associated with metastatic disease at diagnosis. Men were less likely to present with metastatic disease at the time of diagnosis as compared to women, with an OR of 0.60 (95% CI [0.48-0.73], p < 0.01). Compared with CA, MA, and SC were more likely to present with metastasis at diagnosis (OR: 2.34 [1.80- 3.06]; OR 1.93 [1.29-2.89], respectively). GC was less likely (OR: 0.59 [0.36-0.93]). Race and insurance status had no association.

**Table 2:** Factors associated with metastasis at diagnosis in appendiceal carcinoma.

	Firth logistic regression	
	OR (95% CI)	p-value
Age		
<40	Reference	
40-59	1.00 (0.68-1.48)	0.98
60-79	0.85 (0.58-1.25)	0.40
≥80	0.48 (0.29-0.79)	<0.01
Sex		
Female	Reference	
Male	0.60 (0.48-0.73)	<0.01
Race		
White	Reference	
Black	1.09 (0.78-1.51)	0.61
Asians and American Indians	0.89 (0.62-1.29)	0.55
Marital status		
Married	Reference	
Unmarried	0.79 (0.63-0.97)	0.03
Insurance status		
Yes	Reference	
No	1.03 (0.60-1.73)	0.93
Grade		
I	Reference	
II	0.93 (0.73-1.19)	0.57
III	1.61 (1.16-2.24)	<0.01
IV	1.55 (0.87-2.76)	0.14
Histologic Type		
Colonic Adenocarcinoma	Reference	
Goblet Cell/Adenocarcinoid	0.59 (0.36-0.93)	0.02
Mucinous Adenocarcinoma	2.34 (1.80-3.06)	<0.01
Signet Ring Cell Carcinoma	1.93 (1.29-2.89)	<0.01
T Stage		
T1	Reference	
T2	0.34 (0.16-0.71)	<0.01
T3	0.55 (0.34-0.91)	0.02
T4	3.46 (2.24-5.51)	<0.01
N Stage		
N0	Reference	
N1	1.17 (0.88-1.56)	0.29
N2	2.19 (1.53-3.16)	<0.01

Patterns of metastasis at diagnosis

Patterns of metastasis at diagnosis are displayed in Table 3. A total of 90.1% of CA, 84.2% of GC, 42.2% of MA, and 78.5% of SC patients with metastasis at diagnosis had extraperitoneal distant metastasis (M1b). The liver was a more common site of metastasis at diagnosis than lung and bone.

**Table 3:** Patterns of metastasis in newly diagnosed appendiceal carcinoma based on histologic subtype.

	Intraperitoneal metastasis (M1a)	Nonperitoneal distant metastasis (M1b)	Site of distant metastasis				Median survival in months (interquartile range) among patients with metastases
	Number of patients		Liver	Lung	Bone	Brain	
Colonic Adenocarcinoma	20 (9.9%)	183 (90.1%)	68	12	8	0	19.0 (15.9-22.1)
Goblet Cell Adenocarcinoma	9 (15.8%)	48 (84.2%)	6	1	0	0	27.0 (17.2-36.8)
Mucinous Adenocarcinoma	465 (57.8%)	339 (42.2%)	101	27	9	3	60.0 (N/A)*
Signet Ring Cell Carcinoma	39 (21.5%)	142 (78.5%)	7	4	6	0	20.0 (16.8-23.2)
Total	533 (42.8%)	712 (57.2%)	182	44	23	3	35.0 (31.3-38.7)
*Confidence interval could not be calculated since an insufficient number of deaths were observed to estimate a standard error for the median survival time.							

## Discussion

Prior data on metastatic appendiceal carcinoma was insufficient, owing to the rarity of the disease. The current study clarifies the frequency of metastasis in newly diagnosed appendiceal carcinoma, analyzes potential risk factors associated with its presence, and describes the median survival in this population. Knowledge of this epidemiological data may help us appreciate the aggressive behavior of this disease.

Our study noted that a significant percentage of patients were found to have metastatic disease at diagnosis. Unfortunately, early-stage appendiceal carcinoma remains a diagnostic challenge, since patients may not exhibit any symptoms until the tumor is advanced enough to cause blockage or appendicitis. Thus, it is often found incidentally during diagnostic imaging or surgery. When peritoneal dissemination occurs, patients may present with increasing abdominal girth and non-specific abdominal discomfort. Patients with MA can develop mucinous ascites, also known as pseudomyxoma peritonei, which could lead to intestinal obstruction. The clinical presentation of nonperitoneal distant metastases depends on the organ involved. For instance, liver and bone involvement may result in impaired liver function and pathological fractures, respectively.

The most prevalent subtype in our patient cohort was MA. It showed the highest odds of metastasis in newly diagnosed appendiceal carcinoma, when compared to CA. Over half of MA patients had metastasis, and peritoneal dissemination was more common than distant spread. Appendiceal MA with distant metastases is associated with high (50%) five-year survival [5]. However, the high prevalence of metastatic disease is still worrisome. Therapy for the peritoneal disease involves cytoreductive surgery (CRS) in combination with hyperthermic intraperitoneal chemotherapy (HIPEC) if complete tumor debulking is possible, which is inherently aggressive [9]. The combined modality therapy of CRS and HIPEC is associated with perioperative mortality between 0% and 18% and morbidity between 30% and 70% [10]. For women of childbearing age, fertility preservation options need to be considered prior to these treatments. Bilateral oophorectomy is often recommended for postmenopausal women since ovarian metastasis is common, and this may increase the risk of osteoporosis and postmenopausal symptoms [11-13].

Compared to other subtypes, GC had the lowest percentage and odds of metastasis at diagnosis. The vast majority (84.2%) had metastasis beyond the peritoneum (M1b). Like MA treatment, the common practice is to perform CRS and HIPEC for peritoneal involvement. For appendiceal GC patients who receive these treatments, the disease-free and overall survival rates are reportedly 43% and 63%, respectively [3]. For patients with extraperitoneal distant involvement, current practice consists of systemic chemotherapy using 5-flourouracil-based regimens similar to colon cancer treatment.

Appendiceal SC is known to be very aggressive and confers a significantly worse prognosis than other tumor histologies [5]. We found that over one-half cases presented with metastatic disease, and SC had almost twice the odds of metastasis at diagnosis than CA. Studies have reported the five-year cancer-specific survival of metastatic appendiceal GC to be 4-7%, and a median survival of only 19 months, in contrast to 60 months for nonmetastatic disease [4,14]. Our study found the median survival of metastatic disease to be similar at 20 months. The benefit of CRS and HIPEC in appendiceal SC patients with peritoneal dissemination is controversial since prior studies demonstrated inconsistent results [15,16].

About one-quarter of patients with appendiceal CA had metastasis at diagnosis. Most (90.1%) had nonperitoneal distant metastases, of which liver involvement was more common than lung, bone, and brain. The term “colonic-type” is used since it develops from preexisting adenomas similar to colorectal tumors [9]. Treatment of peritoneal-only metastatic disease involves CRS and HIPEC. Surgical resection of metastatic lesions can be performed in certain patients. Systemic chemotherapy can be administered to asymptomatic patients with distant metastasis. The median survival for metastatic appendiceal CA was only 19 months in our study.

Interestingly, we found that men had much lower odds of presenting with metastatic disease. The authors of this study are uncertain why this would be the case. As expected, T4 and N2 stages had much higher odds of metastasis at diagnosis compared to T1 and N0 stages, respectively. Surprisingly, T2 and T3 stages were associated with lower odds of metastasis at diagnosis than the T1 stage. Thus, increasing T and N stages were not necessarily correlated with higher odds of metastatic disease at diagnosis.

There are certain limitations to our study that should be noted. Information on the size or number of metastasis was not available in SEER. Neither was data on several sites of metastasis, such as the ovaries. Information on patient comorbidities was not accessible and may have inadvertently influenced estimations of median survival.

## Conclusions

In summary, this study examined the frequency of metastatic disease in newly diagnosed appendiceal carcinoma. A significant proportion of patients presented with metastatic disease, concerning substantial diagnostic delay and potentiating the need for aggressive treatments. Predictors of metastatic disease included female sex, histologic subtype, and significant regional lymph node involvement. However, increasing primary tumor extent and size was not always predictive. Future research should focus on earlier detection and explore tumor biology.
